# Long noncoding RNA GK‐IT1 promotes esophageal squamous cell carcinoma by regulating MAPK1 phosphorylation

**DOI:** 10.1002/cam4.4795

**Published:** 2022-05-24

**Authors:** Xin Yang, Tianyang Zeng, Ziyang Liu, Wanlun He, Mengting Hu, Ti Tang, Li Chen, Lei Xing

**Affiliations:** ^1^ Department of Thoracic Surgery The First Affiliated Hospital of Chongqing Medical University Chongqing China; ^2^ The Frist People's Hospital Chongqing Liang Jiang New Area Chongqing China; ^3^ Department of Cell Biology and Genetics Chongqing Medical University Chongqing China; ^4^ Department of Endocrine and Breast Surgery The First Affiliated Hospital of Chongqing Medical University Chongqing China

**Keywords:** ERK/MAPK pathway, esophageal squamous cell carcinoma, long noncoding RNA GK‐IT1, malignant progression, MAPK1

## Abstract

**Background:**

Long noncoding RNAs (lncRNAs) are implicated in the oncogenesis and metastasis of multiple human cancers. Nonetheless, the precise molecular mechanisms underlying the oncogenic role of lncRNA in esophageal squamous cell carcinoma (ESCC) remains to be clarified.

**Methods:**

The expression of GK intronic transcript 1 (GK‐IT1) was analyzed using ESCC RNA‐seq data from The Cancer Genome Atlas database. Quantitative real‐time PCR was used to measure the expression of GK‐IT1 in ESCC clinical samples and cells. The correlation between GK‐IT1 expression and clinicopathological variables was examined using chi‐squared tests. Kaplan–Meier survival and Cox regression analyses were employed to generate the survival curve and assess the prognostic value of GK‐IT1. Functional experiments were utilized to explore the role of GK‐IT1 in promoting cell migration, invasion, proliferation, and suppressing apoptosis and autophagy in ESCC. To understand the mechanism, an RNA pulldown assay, RNA immunoprecipitation, agarose gel electrophoresis, immunofluorescence, and co‐immunoprecipitation assays were used.

**Results:**

In this study we identified an unreported lncRNA, termed GK‐IT1 that was aberrantly overexpressed in ESCC tissues and cells. GK‐IT1 was closely associated with advanced clinical stage, and it was an independent prognostic indicator of ESCC. Functional assays verified that GK‐IT1 significantly promoted ESCC proliferation, invasion, and migration, and suppressed ESCC apoptosis and autophagy. Furthermore, tumorigenesis experiments in nude mice indicated that GK‐IT1 promoted ESCC tumor growth and metastasis. Mechanistically, GK‐IT1 competitively bound to mitogen‐activated protein kinase 1 (MAPK1) to prevent the interaction between dual specificity phosphatase 6 (DUSP6) and MAPK1, thereby controlling the phosphorylation of MAPK1 and promoting ESCC progression.

**Conclusion:**

Our study revealed that GK‐IT1 competed with DUSP6 to attenuate the interaction between DUSP6 and MAPK1, leading to activation of the ERK/MAPK pathway, thereby promoting progression of ESCC. Our research indicated that GK‐IT1 served as a novel potential target for the diagnosis and treatment of ESCC.

## Graphical abstract

In this study, we showed that lncRNA GK‐IT1 promotes migration and growth, and inhibits apoptosis of ESCC cells both in vivo and in vitro. Mechanistic investigations further revealed that lncRNA GK‐IT1 could competitively bind to MAPK1 to prevent the interaction between DUSP6 and MAPK1, facilitating activation of ERK/MAPK pathway and ultimately promoting progression of ESCC.

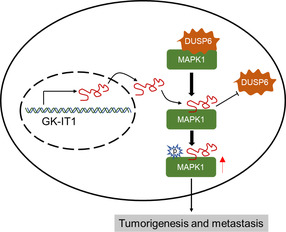

## 1 INTRODUCTION

Esophageal cancer (ESCA) is one of the most aggressive malignant tumors, imposing a serious threat to public health worldwide.^1^ In particular, esophageal squamous cell carcinoma (ESCC) is the major histological subtype of ESCA.^2^ Despite the fact that surgery, radiotherapy and chemotherapy have improved the possibility for recovery, a large proportion of patients with metastatic ESCA still exhibit poor survival rates.^3^, ^4^, ^5^ Thus, further investigation is warranted to comprehensively understand the underlying mechanisms of ESCC progression.

LncRNAs belong to a class of RNA molecules that comprise more than 200 nucleotides and possess nonprotein‐coding capacity.^6^ However, because of their general low abundance and modest evolutionary conservation compared to protein‐coding genes, the biological roles and the related molecular mechanisms for the majority of lncRNAs remain unexplored.^7^ Accumulating research has revealed that lncRNAs play extensive roles in facilitating the development of numerous diseases and human cancers.^8^, ^9^, ^10^ The most commonly studied functions of lncRNA are regulating gene transcription, such as recruiting chromatin‐modifying complexes,^11^ and serving as competitive endogenous RNAs.^12^ For example, the lncRNA SChLAP1 antagonizes the SWI/SNF complex, a major epigenetic complex, to modulate gene expression in prostate cancer.^13^ LncARSR acts as a competitive RNA (ceRNA) for miR‐34 and miR‐449 to promote AXL and c‐MET expression, causing sunitinib resistance and predicting poor response of renal cancer patients.^14^, ^15^ LINC00908 may act as a competing endogenous RNA to facilitate the expression of the miR‐143‐3p target gene *KLF5* in colorectal cancer.^16^ Recent research has reported that lncRNAs could interact with proteins to exert biological functions.^17^ For instance, lncRNA BS‐DRL1 can interact with HMGB1, thus modulating the DNA damage response.^7^ LncRNA UFC1 increases levels of β‐catenin in hepatocellular carcinoma by binding to human antigen R (HuR).^18^ The RNA binding protein PABPC1 can induce lncRNA BDNF‐AS to inhibit the malignant progression of glioblastoma. Nevertheless, the interaction between lncRNAs and proteins in ESCC is not well understood.

Dysregulation of the extracellular signal‐regulated kinase/mitogen‐activated protein kinase (ERK/MAPK) signaling pathway has been widely implicated in a range of human diseases, including cancers.^19^, ^20^ Canonically, upstream kinases mediate the activation of the ERK/MAPK pathway, which may further regulate many cellular biological processes, including proliferation, differentiation, and transformation.^21^ In addition, an increasing amount of evidence has indicated that lncRNAs can also contribute to the activation of the ERK/MAPK pathway. For instance, UBE2CP3 promotes angiogenesis in hepatocellular carcinoma by activating the ERK/MAPK pathway.^22^ Huang et al. reported that the hepatitis B virus X protein (HBx)‐related lncRNA DBH‐AS1 activated the ERK/MAPK pathway, which promoted cell proliferation and survival in hepatocellular carcinoma.^23^ It is well‐known that DUSP6 can dephosphorylate and inactivate ERK1/2. The study has shown that PLAC8 directly bound DUSP6 and increased the phosphorylation of MAPK1, thus promoting the epithelial‐mesenchymal transition (EMT) in colon cancer.^24^ Follicle‐stimulating hormone (FSH) can regulate MAPK1 phosphorylation by inactivating DUSP6 in granulosa cells.^25^ miR‐181a can inhibit the expression of DUSP6 to regulate the ERK/MAPK pathway.^26^ However, our understanding of lncRNAs directly interacting with MAPK1 protein to regulate the cellular biological processes in human cancers remains poorly studied.

In this study, we analyzed RNA‐seq data from The Cancer Genome Atlas (TCGA), and investigated the lncRNA expression profile in ESCC tissues. We focused on the novel lncRNA GK‐IT1, examining its biological functions as well as the underlying molecular mechanisms of ESCC development. We found that lncRNA GK‐IT1 was significantly overexpressed in ESCCs and closely associated with advanced ESCC Tumor, Node, Metastasis (TNM) stage, and poor patient survival. We showed that lncRNA GK‐IT1 promoted migration and growth and inhibited apoptosis of ESCC cells both in vivo and in vitro. Mechanistic explorations further revealed that lncRNA GK‐IT1 could competitively bind to MAPK1 to prevent the interaction between DUSP6 and MAPK1, facilitating activation of the ERK/MAPK pathway to promote ESCC progression.

## 2 MATERIALS AND METHODS

### 2.1 Human ESCC tissue specimens

ESCC tissue specimens used in this study were provided by 70 patients who underwent surgical resection and had no history of chemotherapy or immunotherapy prior to surgery at The First Affiliated Hospital of Chongqing Medical University (Chongqing, China). These tissue samples were immediately stored in liquid nitrogen upon collection. All patients signed their informed consent forms, and the study was authorized by the Ethics Committee of Chongqing Medical University.

### 2.2 Bioinformatics analysis

ESCA RNA‐seq data were downloaded from the TCGA (https://portal.gdc.cancer.gov/) database, which included 162 cancer tissue samples and 11 adjacent normal tissue samples. The HTSeq‐counts data in the database were analyzed using R software (https://www.r‐project.org). According to the screening criteria set at a fold change >2, *p* < 0.05, and FDR < 0.01, 340 upregulated and 259 downregulated lncRNAs were identified. A Kaplan–Meier survival analysis was conducted to assess the difference in the survival of ESCA patients between GK‐IT1‐high expression and GK‐IT1‐low expression patients. Patients with a history of chemotherapy or other therapies were excluded, and the remaining patients were divided into two groups according to the median expression of GK‐IT1. In addition, a univariate Cox analysis was employed to test the prognostic value of GK‐IT1 in ESCC.

### 2.3 Cell lines and culture conditions

Five human esophageal squamous cell lines (TE‐1, TE‐10, ECA‐109, Kyse‐510, and Kyse‐150) and a normal esophageal epithelial cell line (Het‐1a) were purchased from the ATCC (American Type Culture Collection). The TE‐10, TE‐1, Het‐1a, ECA‐109, KYSE‐510, KESE‐150, and HEK‐293T cells were all cultured in DMEM medium (Gibco). The cell culture medium contained 10% fetal bovine serum (HyClone, Invitrogen), 100 U/ml penicillin, and 100 mg/ml streptomycin. Cells were cultured in a humidified incubator containing 5% CO_2_ at 37°C.

### 2.4 Plasmid construction, RNAi, and cell transfection

To construct the GK‐IT1 overexpression plasmid, full‐length human GK‐IT1 was inserted into the pcDNA3.1‐EF1a‐mcs‐3flag‐CMV‐GFP vector (Shanghai, China, Hanbio) and the sequence without GK‐IT1 (vehicle) was used as the control. Small interfering RNAs (siRNAs) against GK‐IT1 (siGK‐IT1‐1, siGK‐IT1‐2, and siGK‐IT1‐3) were synthesized to knockdown GK‐IT1, and a scrambled si‐NC (Genechem Biotech) sequence was used as a control. The most effective siRNA siGK‐IT1‐2 and GK‐IT1 overexpression plasmid were subcloned into a lentiviral vector (hU6‐MCS‐Ubiquitin‐firefly_Luciferase‐IRES‐puromycin) to construct a sh‐GK‐IT1 carrier, and sh‐NC was used as a negative control (Guangzhou, Genechem Biotech). The two helper plasmids as well as a lentiviral plasmid carrying either GK‐IT1 or sh‐NC were transfected into HEK293T cells. The viral supernatant was collected and centrifuged for animal research 72 h after transfection. Puromycin was then used to select stably transfected or infected ESCC cells. The sequences of siRNAs and shRNAs in this study are listed in File S1: Table S1.

### 2.5 RNA extraction, nuclear‐cytoplasmic fractionation, and quantitative real‐time PCR

Total RNA was isolated from paired tumor and normal tissues using TRIzol reagent (Takara) following the manufacturer's instructions. RNA from the cytoplasm and nucleus of ESCC cells were separated using the PARIS™ Kit (Life Technologies). Quantitative real‐time PCR (qRT‐PCR) analysis was performed using the TB Green Premix Ex Taq (Takara, Japan) and the Biosystems 7900 Real Time PCR System (Thermo Fisher Scientific). Relative expression levels of genes were quantified using the 2^−ΔΔCt^ method. The primer sequences are displayed in Table S1.

### 2.6 Fluorescence in situ hybridization assay

To observe the subcellular localization of GK‐IT1 in ESCC cells and tissues, fluorescence in situ hybridization (FISH) assays were performed using the Ribo Fluorescent In Situ Hybridization Kit (RIOBIO) according to the manufacturer's instructions. ESCC cells were seeded onto cover slides in 24‐well plates and cultured until the cell density reached 60%–70%. Then, cells and frozen sections of ESCC tissues were fixed with 4% paraformaldehyde at room temperatures for 10 min, followed by permeabilization using Triton X‐100 at 4°C for 30 min. Next, the samples were pre‐hybridized with pre‐hybridization solution at 37°C for 30 min and incubated with cy3‐labeled lncRNA, U6, or 18 s FISH Probe Mix at 37°C overnight. Samples were then washed with PBS three times. Next, the cell nucleus was counterstained with 4,6‐diamidino‐2‐phenylindole (RIOBIO).

### 2.7 Cell proliferation assay

A colony formation assay, 5‐ethynyl‐2′‐deoxyuridine (EdU) incorporation assay, and Cell Counting Kit‐8 (CCK‐8) assay were used to detect the proliferative capabilities of ESCC cells. For the colony formation assay, 350 transfected cells/well were inoculated into 6‐well plates and cultured for 2 weeks. The colonies were then fixed with 4% paraformaldehyde and stained with 0.5% Crystal Violet to facilitate counting of the total number of colonies.

The EdU assay was performed following the manufacturer's instructions (RIOBIO). Cells were seeded into 24‐well plates, incubated with 50 μM EdU‐containing culture media for 2 h and washed twice with PBS. Cells were fixed with 4% paraformaldehyde and stained with Apollo® fluorescent dye, both of which were incubated at room temperatures for 30 min. The nuclei were stained with Hoechst® 33,342 at room temperature for 30 min.

For the CCK‐8 assay, 100 μl of cells (1 × 10^5^/ml) from each group was inoculated into 96‐well plates in replicates 24 h post‐transfection. Then, 10 μl of CCK8 reagent (Beyotime) was added into each well, and cells were incubated at 37°C for 2 h in the dark. The OD value (450 nm) was then measured using a spectrophotometer (Thermo Fisher).

### 2.8 In vitro cell migration and invasion assay

Horizontal cell migration was examined using a wound healing assay. When cell confluency reached approximately 95%, media was removed and a perpendicular scratch across the monolayer was made using a 10 μl pipette tip. Cells were washed three times with PBS and cultured in serum‐free media. Using an inverted microscope (Leica), the cells were photographed at 0 and 24 h after wounding. Cell migration capabilities were evaluated by assessing the width of the remaining scratch under the microscope. For cell invasion assays, the transfected cells were resuspended in serum‐free media at a concentration of 7.5 × 10^4^ cells/ml, then 500 μl per well of complete media was added into 24‐well plates. Transwell chambers were precoated with Corning Matrigel (BD Bioscience; cat. no. 354234) 6 h prior to the experiment and placed into the well. Then, 200 μl of premixed cell suspension was added to the wells. The cells were then incubated at 37°C in normal cell culture conditions for 16–20 h. Cells that invaded through the chamber bottom were fixed with methanol for 20 mins at room temperature and stained with 0.1% (w/v) Crystal Violet for 5 min.

### 2.9 Flow cytometry analysis

For cell cycle analysis, cells were harvested 72 h after transfection and fixed in pre‐cooled 70% ethanol at 4°C overnight. Cells were then stained with propidium iodide (PI), and detected using flow cytometry (Becton Dickinson FACS Calibur). For the apoptosis assay, cells were collected and resuspended in 500 μl of cold PBS after centrifugation. Annexin V‐FITC and PI staining was then carried out followed by flow cytometry to measure the cell apoptosis rate.

### 2.10 Terminal deoxynucleotidyl transferase dUTP nick‐end labeling assay

A terminal deoxynucleotidyl transferase dUTP nick‐end labeling (TUNEL) assay was used to analyze apoptosis in TE‐1 and TE‐10 cells. Cells were seeded into 24 well plates for 72 h, washed with PBS, and fixed with 4% paraformaldehyde for 1 h. Next, the cells were incubated in a 0.3% Triton X‐100/PBS mixture at room temperature for 5 min and then in detection solution for 1 h at 37°C in the dark. Images were captured under a fluorescence microscope (Leica).

### 2.11 RNA pulldown and mass spectrometry analysis

A Pierce™ Magnetic Ribonucleic Acid‐Protein Pulldown Kit (Thermo Fisher) was used for RNA pulldown and liquid chromatography‐mass spectrometry (LC–MS) analysis following the manufacturer's instructions. Briefly, biotin‐labeled antisense RNA and sense RNA (Invitrogen) were used to prepare biotin‐labeled GK‐IT1 according to the manufacturer's instructions. The biotinylated GK‐IT1 RNA was incubated with streptavidin beads and total cell lysate for 2 h, then centrifuged at 4°C for 5 min. Next, the RNA‐protein complex was eluted with 1× SDS loading buffer. The enriched proteins were separated in a 10% SDS‐PAGE gel and the gel was sliver stained. The protein bands were cut from the gel and analyzed by LC–MS. Common contaminating proteins including keratin, antibody proteins, and serum albumin were eliminated by setting the criteria of confidence value ≥95% and including at least one unique peptide.

### 2.12 RNA immunoprecipitation

The RNA immunoprecipitation (RIP) assay was performed with a Magna RIP Kit (Millipore), following the manufacturer's protocols. Briefly, the magnetic beads were incubated with anti‐MAPK1 antibodies (Abcam) or IgG negative control antibodies (Millipore). Then, cells were lysed and incubated with corresponding antibody‐coated beads. Subsequently, the coprecipitated RNA was extracted using TRIzol reagent (Takara). RNA levels were detected using qRT‐PCR and agarose gel electrophoresis.

### 2.13 Immunohistochemistry and immunofluorescence

Immunohistochemistry (IHC) and immunofluorescence (IF) assays were performed as previously reported.^27^ For the IF experiment, ESCC cells were incubated with primary antibodies against LC3B (1:200 dilution) (Cell Signaling Technology), MAPK1 (1:200) (Cell Signaling Technology), and p‐MAPK1 (1:200) (Cell Signaling Technology) at 4°C overnight. Cells were then incubated with fluorescein‐conjugated secondary antibodies (Alexa Fluor 488) at room temperature for 1 h. Coverslips were imaged using a fluorescence microscope (Leica).

For IHC analysis, xenograft tumors were immediately immersed in 4% paraformaldehyde after resection for 24 h and used to produce paraffin sections. Following dewaxing and hydration, slides were incubated with 3% H_2_O_2_ to block endogenous peroxidase and then heated in 0.01 M sodium citrate for 10 min for antigen retrieval. Next, the tissues were covered with primary antibodies against p‐MAPK1 (1:200) and MAPK1(1:200) (Cell Signaling Technology) at 4°C overnight, followed by incubation with fluorophore‐conjugated secondary antibodies (Alexa Fluor 488) at room temperature for 1 h. The slides were first stained with DAB and then counterstained with hematoxylin. The images were photographed under an Olympus multifunction microscope (Olympus BX51).

### 2.14 Co‐immunoprecipitation (Co‐IP) assay

TE‐10 cells were transfected with lncRNA GK‐IT1‐overexpressing plasmids and siGK‐IT1‐2 with Lipofectamine 2000 (Thermo Fisher Scientific). The co‐immunoprecipitation assay was carried out using the Pierce Classic Magnetic IP/Co‐IP Kit (Thermo Scientific) following the manufacturer's protocols. In brief, cells were harvested, lysed using cold IP lysis/wash buffer, and centrifuged at 13,000 × *g* for 10 min. Supernatants were obtained and then incubated with 10 μg IgG or anti‐MAPK1 antibody‐coated beads (Thermo Fisher Scientific) and mixed at 4°C overnight. The beads were washed with IP lysis/wash buffer twice, with purified water once, and then subject to elution of the antibody–antigen complexes. Western blot assays were carried out to detect DUSP6 and MAPK1 protein.

### 2.15 Western blot analysis

In short, proteins were extracted from ESCC cells and tissues, quantified using the BCA assay, and separated on a 10%–12% SDS‐PAGE gel. After electrophoresis, the protein bands were transferred to PVDF membranes (Bio‐Rad). The membranes were then blocked with 5% skim milk at room temperature for 1 h and incubated with primary antibodies against the following proteins overnight at 4°C: MAPK1 (1:1000) (Santa Cruz Biotechnology), MEK1/2 (1:1000), p‐MEK1/2 (1:1000), p‐MAPK1 (1:1000) (CST, Cell Signaling Technology), Cyclin E1 (1:1000) (Abcam), Bax (1:1000, CST), Bcl‐2 (1:1000, CST), cleaved caspase‐3 (1:1000, CST), LC3B (1:1000, CST), ATG5 (1:1000, CST), ATG7 (1:1000, CST), Cyclin D1 (1:1000, CST), CDK4 (1:1000, CST), DUSP6 (1:1000, CST), and GAPDH (1:5000, Abcam). The blots were then incubated with secondary antibodies (1:5000, CST) for 1 h at room temperature. Finally, images of the bands were visualized using a chemiluminescence system (Bio‐Rad).

### 2.16 Animal experiments

For the animal experiments, 4‐week‐old female BALB/c nude mice were used for xenotransplantation experiments and kept under specific pathogen‐free conditions. All studies were approved by the Animal Protection and Utilization Committee of Chongqing Medical University. A total of 200 μl of suspension of TE‐10 cells stably transfected with GK‐IT1 overexpressing/mock vector or infected with lentivirus carrying sh‐GK‐IT1/sh‐NC (Genechem Biotech) (1.25 × 10^6^/ml) were randomly subcutaneously inoculated into nude mice. Mice were sacrificed 4 weeks later. The tumors were measured once a week, and tumor volume was calculated according to the formula: length×width^2^/2. Tumor weights were also measured. Lung and liver tissues were collected and made into paraffin sections for hematoxylin and eosin staining. For the hematoxylin and eosin‐stained tumor sections, liver metastatic nodules were counted under a microscope. For the survival analysis, cells stably transfected with GK‐IT1 or mock vector were subcutaneously injected into mice. The cutoff point for monitoring the tumors was 2 months. Sixty days later, the remaining mice were examined. The mice were then sacrificed, and their livers were taken for pathological analysis.

### 2.17 Statistical analysis

Data were presented as the mean ± SD. The differences between two groups were compared using two‐tailed Student's *t*‐test or chi‐squared test. To analyze the differences between groups, ANOVA test was used. Survival rates were evaluated using the Kaplan–Meier analysis and then compared using the log‐rank test. The hazard ratio (HR) and 95% confidence interval (CI) were calculated using the Cox proportional hazards model with a *p* < 0.05 being considered statistically significant. All experiments in this study were independently repeated for three times. Statistical analyses were conducted using SPSS 21.0 (IBM, SPSS) and GraphPad Prism 6.0 (GraphPad Software Inc.).

## 3 RESULTS

### 3.1 LncRNA GK‐IT1 is overexpressed in ESCC and associated with tumor progression

**FIGURE 1:**
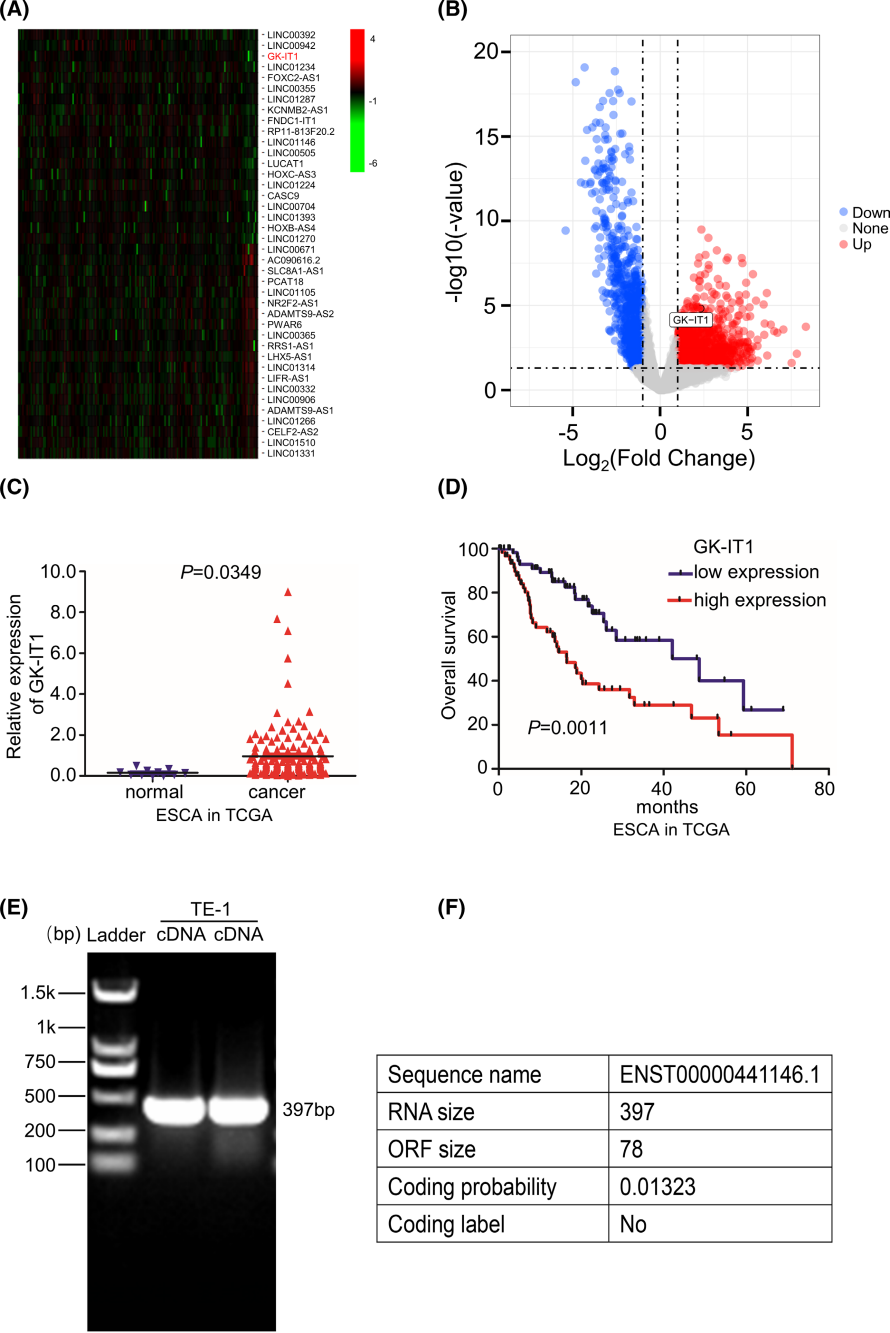
GK‐IT1 is upregulated in esophageal cancer tissues and associated with poor prognosis. (A, B) The heat map and scatter map were obtained through the differential expression analysis of RNAs in esophageal cancer in TCGA database. (C) GK‐IT1 expression was detected in ESCA based on data derived from TCGA database. (D) Kaplan–Meier survival analysis of the overall survival in two groups stratified by low and high expression of GK‐IT1 in ESCC patients based on clinical data derived from TCGA database. (E) The existence of GK‐IT1 from cDNA in TE‐1 cell was detected by RT‐PCR. GK‐IT1 could be amplified from cDNA of TE‐1. (F) The protein‐coding potential of GK‐IT1 was analyzed using CPC 2.0 online website. The median expression of GK‐IT1 was used as cutoff *p* = 0.0011 by log‐rank test

**FIGURE 2:**
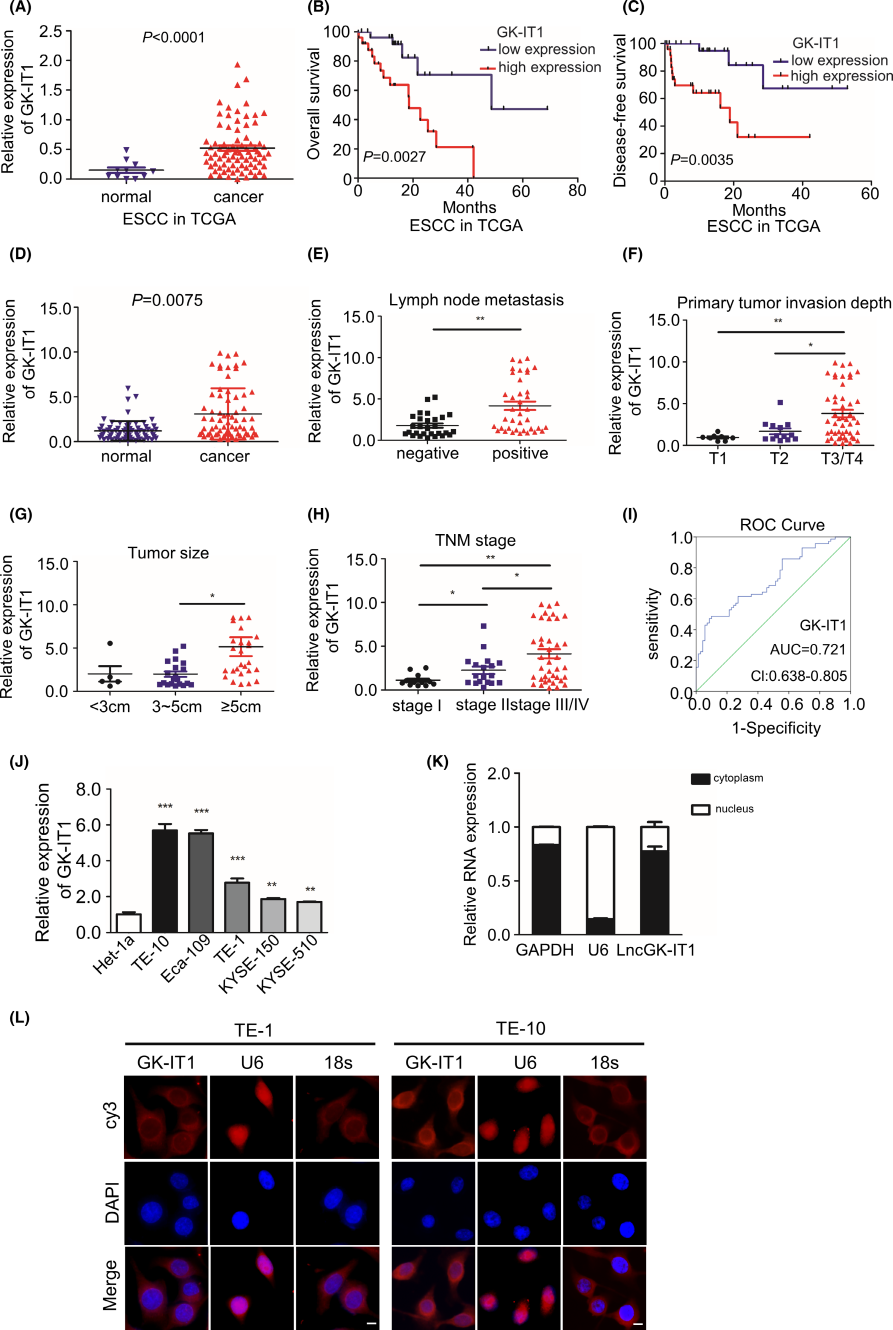
GK‐IT1 is upregulated in ESCC tissues and associated with clinical progression. (A) GK‐IT1 expression was detected in ESCC. (B, C) Kaplan–Meier survival analysis of the overall survival and disease‐free survival in two groups defined by low and high expression of GK‐IT1 in patients with ESCC. The median expression of GK‐IT1 was used as cutoff. *p* = 0.0027 and *p* = 0.0035 by log‐rank test. (D) Comparison of GK‐IT1 expression in ESCC patient's tissues (*n* = 70) and normal tissues (*n* = 70) by qRT‐PCR. (E–H) The expression of GK‐IT1 was determined by qRT‐PCR at different primary tumor invasion depth, lymph node metastasis, TNM stages, Tumor size, of ESCC patients compared with corresponding adjacent normal tissues. (I) ROC curve was applied to evaluate the diagnostic value of GK‐IT1 for ESCC patients. (J) Relative expression of GK‐IT1 in five ESCC cell lines (TE‐10, TE‐1, Eca‐109, KYSE‐150, and KYSE‐510) and an esophageal epithelial cell (Het‐1a). (K) Nuclear‐cytoplasmic fractionation assay indicated that lncRNA GK‐IT1 was mainly localized in the cytoplasm of ESCC cells. (L) The localization of GK‐IT1was observed in ESCC cells (magnification, ×400, Scale bar, 20 μm) by FISH. The nuclear was stained with DAPI. The data are presented as the mean ± SD, **p < 0.01, ***p < 0.001

To determine the differentially expressed lncRNAs in esophageal tumorigenesis, we analyzed RNA‐seq data obtained from TCGA database. With the threshold setting as fold change >2 and *p* < 0.05, a total of 340 lncRNAs were aberrantly upregulated. GK‐IT1 was the top upregulated lncRNA that predicted ESCA patient outcome (Figure 1A–D). GK‐IT1, the length of which is 397, is a novel lncRNA located on human chromosome X: 30,671,635‐30,672,166 that was previously unreported in ESCC. The Ensembl Gene ID and the Transcript ID of GK‐IT1 are ENSG00000229331 and ENST00000441146.1, respectively. The RT‐PCR revealed that GK‐IT1 could be detected in TE‐1 cells (Figure 1E). According to Coding Potential Calculator 2 (CPC 2.0 http://cpc2.gao‐lab.org/), we predicted the coding ability of GK‐IT1 and found that it fit the characteristics of lncRNA (Figure 1F). We then sought to determine if GK‐IT1 could be an ESCC‐associated lncRNA by performing differential expression analysis in TCGA data from 80 ESCC tissues and 11 non‐tumor tissues. Kaplan–Meier survival curves showed that high expression of GK‐IT1 was associated with a worse survival rate and shorter disease‐free survival in ESCC patients (Figure 2A–C). Next, qRT‐PCR was performed to measure the expression of GK‐IT1 in 70 pairs of clinical ESCC tissues and adjacent normal tissues. The results further confirmed that the expression of GK‐IT1 was significantly higher in ESCC tissues than that in adjacent normal tissues (Figure 2D). In addition, the expression of GK‐IT1 was positively associated with the depth of tumor invasion, lymph node metastasis status, TNM staging, and tumor sizes (Figure 2E–H). A receiver operating characteristic curve was further utilized to assess the diagnostic value of GK‐IT1 for ESCC. The area under the curve of GK‐IT1 was 0.721, suggesting that it could sensitively discriminate ESCCs (Figure 2I). Moreover, the upregulation of GK‐IT1 expression was confirmed in ESCC cell lines (TE1, TE10, Eca109, KYSE150, and KYSE510) as compared to normal esophageal epithelial cells (Het‐1a) by qRT‐PCR (Figure 2J). We next evaluated the relationship between GK‐IT1 expression and clinicopathological features from TCGA database. As shown in Table 1, the expression of GK‐IT1 was positively correlated with T stage (*P* = 0.004) and N stage (*P* = 0.030) (Table 1). Furthermore, we validated the clinical relevance of GK‐IT1 in ESCC using the clinical information of ESCC patients from TCGA database and found that GK‐IT1 expression was positively associated with T stage (*P* = 0.032) and N stage (*P* = 0.04) in ESCC patients (Table 2). By adopting the Cox proportional hazard model and found that GK‐IT1 was likely to be a potential independent predictor for poor prognosis in ESCC patients (HR = 3.329, *P* = 0.026) (Table 3). The subcellular localization of GK‐IT1 in ESCC cells and tissues was predicted using the lncLocator website (http://www.csbio.sjtu. edu.cn/bioinf/lncLocator/#) (Figure S1A). Nuclear‐cytoplasmic fractionation and FISH assays were performed. GK‐IT1 was mainly distributed in the cytoplasm of ESCC (Figure 2K,L and Figure S1B). Taken together, the expression of GK‐IT1 was elevated in ESCCs and associated with the malignant progression of ESCC patients.

**TABLE 1:** Correlation between LncGK‐IT1 expression and clinicopathological features in 70 ESCC patients

Characteristic	All cases	LncGK‐IT1		Chi‐square	*p*‐value
		High	Low		
All cases	70				
Age					
<58	13	5	8	0.902	0.342
>58	56	30	26		
Gender					
Male	57	27	30	0.850	0.356
Female	13	8	5		
Grade					
G1/G2	52	24	28	3.447	0.063
G3	15	11	4		
T stage					
T1/2	21	5	16	8.231	0.004**
T3/4	49	30	19		
N stage					
N0	31	11	20	4.690	0.030*
N1/N2/N3	39	24	15		
TNM stage					
I/II	34	15	19	0.915	0.339
III/IV	36	20	16		

*Note*: The correlation between GK‐IT1 expression and clinicopathological variables was examined using chi‐squared tests.

* *p* < 0.05,

** *p* < 0.01

**TABLE 2:** Correlation between LncGK‐IT1 expression and clinicopathological features in 80 ESCC patients

Characteristic	All cases	LncGK‐IT1		Chi‐square	*p*‐value
		High	Low		
*All cases*					
Age					
<58	80			0.853	0.356
>58	30	13	17		
Gender					
Male	50	27	23	2.296	0.13
Female	67	36	31		
BMI					
18.5–23.9	13	4	9	2.257	0.133
<18.5	58	32	26		
>23.9	4	2	2		
Grade					
G1/G2	18	6	12	1.21	0.271
G3	51	29	22		
T stage					
T1/2	19	8	11	4.575	0.032*
T3/4	35	13	22		
N stage					
N0	44	27	17	4.192	0.04*
N1/N2/N3	43	17	26		
TNM stage					
I/II	35	22	13	1.075	0.3
III/IV	53	24	29		
Alcohol use					
No	26	15	11	3.464	0.063
Yes	21	7	14		
Tobacco use					
No	56	32	24	1.545	0.214
Yes	38	16	22		

*Note*: The correlation between GK‐IT1 expression and clinicopathological variables was examined using chi‐squared tests.

* *p* < 0.05,

** *p* < 0.01.

**TABLE 3:** Univariate and multivariate Cox regression analyses of LncGK‐IT1 and survival in patients with ESCC

Clinical variables	Univariate analysis		*p*‐value
	HR	95% CI	
Age	1.075	0.466–2.479	0.865
Gender	0.227	0.051–1.007	0.051
Tobacco use	2.000	0.825–4.850	0.125
Alcohol use	2.280	0.670–7.755	0.187
Grade (G1 vs G2)	2.606	0.744–9.123	0.134
Grade (G1 vs G3)	1.356	0.687–2.676	0.38
T stage (T1T2 vs T3T4)	1.343	0.593–3.040	0.479
N stage (N0 vs N1N2N3)	2.642	1.071–6.517	0.035*
TNM (I II vs III IV)	3.292	1.404–7.719	0.006**
LncRNA GK‐IT1	3.751	1.363–10.320	0.01*

Clinical variables	Multivariate analysis		*p*‐value
	HR	95% CI	
TNM (I/II vs III/IV)	3.166	0.780–12.842	0.107
N stage (N0 vs N1/N2/N3)	0.848	0.188–3.831	0.83
LncRNA GK‐IT1	3.329	1.152–9.623	0.026*

*Note*: Cox regression analyses were employed to assess the prognostic value of GK‐IT1.

* *p* < 0.05,

** *p* < 0.01.

### 3.2 GK‐IT1 promotes the proliferation, migration, and invasion of ESCC

**FIGURE 3:**
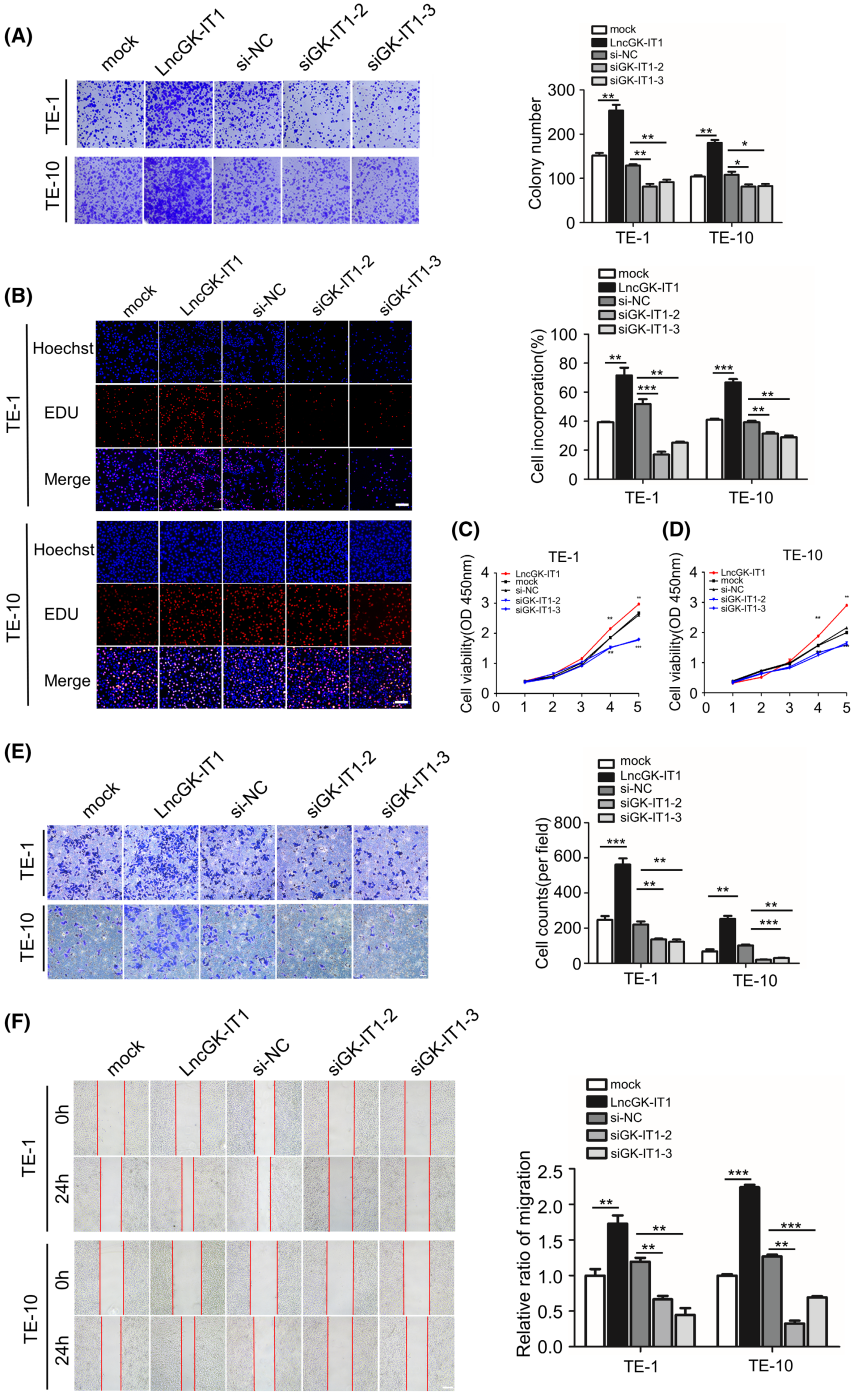
GK‐IT1 affects proliferation, migration, and invasion of ESCC cells. (A) Cell proliferation was evaluated by colony formation assay. (B) Cell proliferation was evaluated by EdU assay (magnification, ×100, Scale bar, 100 μm). (C, D) The growth curves of cells were measured by CCK‐8 assay. (E) The invasive abilities of ESCC cells were assessed by transwell invasion assay (magnification, ×100, Scale bar, 100 μm). (F) The migration abilities of ESCC cells were measured by wound healing assays (magnification, ×50, Scale bar, 100 μm). The data are presented as the mean ± SD, *p < 0.05, **p < 0.01, ***p < 0.001

To probe the potential biological roles of GK‐IT1 in ESCC, we constructed an overexpression plasmid for GK‐IT1 and designed three siRNAs that target the gene sequences of GK‐IT1 to establish GK‐IT1 gain‐of‐function and loss‐of‐function cell models of ESCC, respectively. The infection efficiency of the indicated overexpressing or mimics plasmids and siRNAs or scrambled control was detected using qRT‐PCR (Figure S2A–D). To minimize the chances of off target effects, the two siRNAs exhibiting higher silencing efficiencies were selected for further study. Colony formation, CCK‐8, and EdU assays were carried out for the assessment of cell viability. Silencing of GK‐IT1 notably impaired the viability of ESCC cells, whereas overexpression of GK‐IT1 led to increased ESCC viability (Figure 3A–D). Similarly, we found that depletion of GK‐IT1 inhibited the migratory and invasive capabilities of ESCC cells, whereas increased GK‐IT1 expression enhanced migration and invasion of ESCCs as measured by wound healing and Transwell assays (Figure 3E,F). Collectively, these data suggested that GK‐IT1 facilitates proliferation, migration, and invasion of ESCC cells.

### 3.3 GK‐IT1 regulates cell cycle progression, apoptosis, and autophagy in ESCC

**FIGURE 4:**
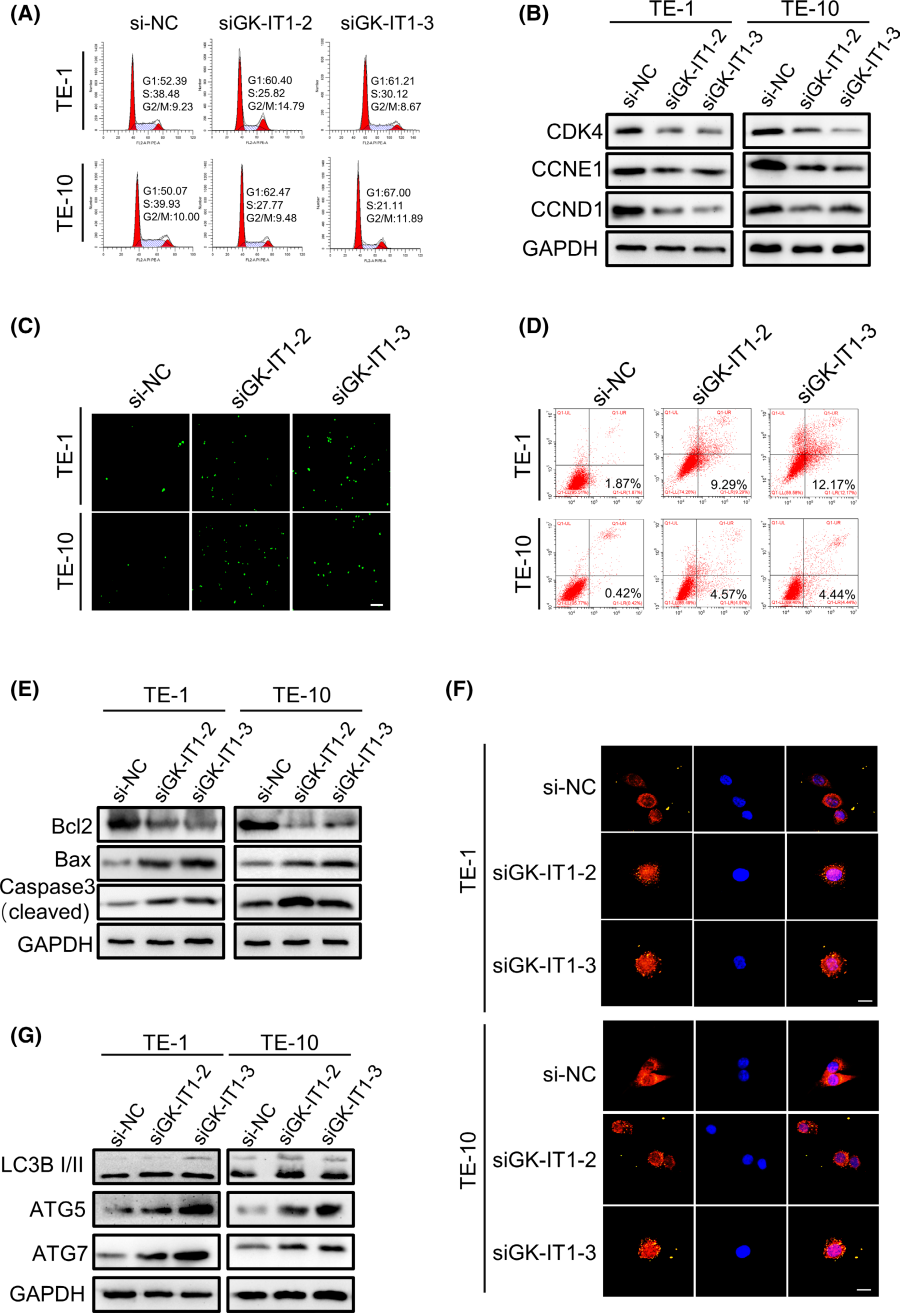
Knockdown of GK‐IT1 induces cell cycle arrest, apoptosis, and autophagy of ESCC cells. (A) Cell cycle analysis was performed using flow cytometry in TE‐1, TE‐10 cells transfected with si‐GK‐IT1. (B) Western blot was conducted to determine the expression of the cell cycle‐related proteins in ESCC cells after transfection with si‐GK‐IT1.(C) Apoptotic ESCC cells were observed with TUNEL method (magnification, ×100, scale bar, 100 μm). (D) Apoptosis rate was detected using flow cytometry after knockdown of GK‐IT1. (E) Western blot was conducted to determine the expression of the cell apoptosis‐related proteins in ESCC cells after transfection with si‐GK‐IT1. (F) The endogenous LC 3B puncta formation was assessed by IF analysis and the total number of LC3B puncta per cell was counted (magnification, ×400, Scale bar, 20 μm). (G) The levels of autophagy‐related proteins were detected by western blot. The data are presented as the mean ± SD, *p < 0.05, **p < 0.01, ***p < 0.001

To assess the effects of GK‐IT1 on ESCC progression, we assessed cell cycle progression, apoptosis, and autophagy. Flow cytometric analysis of cell cycle progression showed that silencing of GK‐IT1 remarkably increased ESCC cell distribution in the G1 phase and reduced distribution in the S phase, which suggests that cell cycle arrest in the G1 phase was induced by knockdown of GK‐IT1 (Figure 4A and Figure S3A,B). Moreover, western blot assays demonstrated that depletion of GK‐IT1 decreased the abundance of cell cycle‐related proteins, further confirming blockade of cell cycle progression by GK‐IT1 knockdown in ESCC cells (Figure 4B and Figure S3C). The effect of GK‐IT1 on apoptosis of ESCC cells was also assessed. Using the terminal deoxynucleotidyl transferase dUTP nick‐end labeling (TUNEL) assay, we found that silencing GK‐IT1 markedly increased the percentage of apoptotic cells in comparison with control groups (Figure 4C). Furthermore, flow cytometric analysis of cell apoptosis through Annexin V/PI double staining displayed the same trend (Figure 4D and Figure S3D). The expression of apoptosis‐related proteins was also determined using western blot. In accordance with the above results, depletion of GK‐IT1 significantly increased expression of proapoptotic proteins including activated (cleaved) caspase‐3 and Bax, but decreased expression of antiapoptotic proteins Bcl‐2 in ESCC cells. On the other hand, overexpression of GK‐IT1 resulted in decreased cleaved caspase‐3 and Bax, and increased Bcl‐2 (Figure 4E and Figure S3E). We next examined the effects of GK‐IT1 knockdown on the autophagic status of ESCC cells. An IF assay showed that depletion of GK‐IT1‐activated cellular autophagy, as evidenced by induction of LC3B‐II puncta and accumulation of autophagosomes (Figure 4F and Figure S3F). Using western blots, we found that knockdown of GK‐IT1 significantly increased ATG5 and ATG7 proteins levels, markers of the conversion from LC3B‐I to LC3B‐II, further supporting that cellular autophagic flux was markedly increased (Figure 4G and Figure S3G). Taken together, these results demonstrated that GK‐IT1 may participate in the regulation of cell cycle progression, apoptosis, and autophagy in ESCC cells.

### 3.4 GK‐IT1 interacts with MAPK1

**FIGURE 5:**
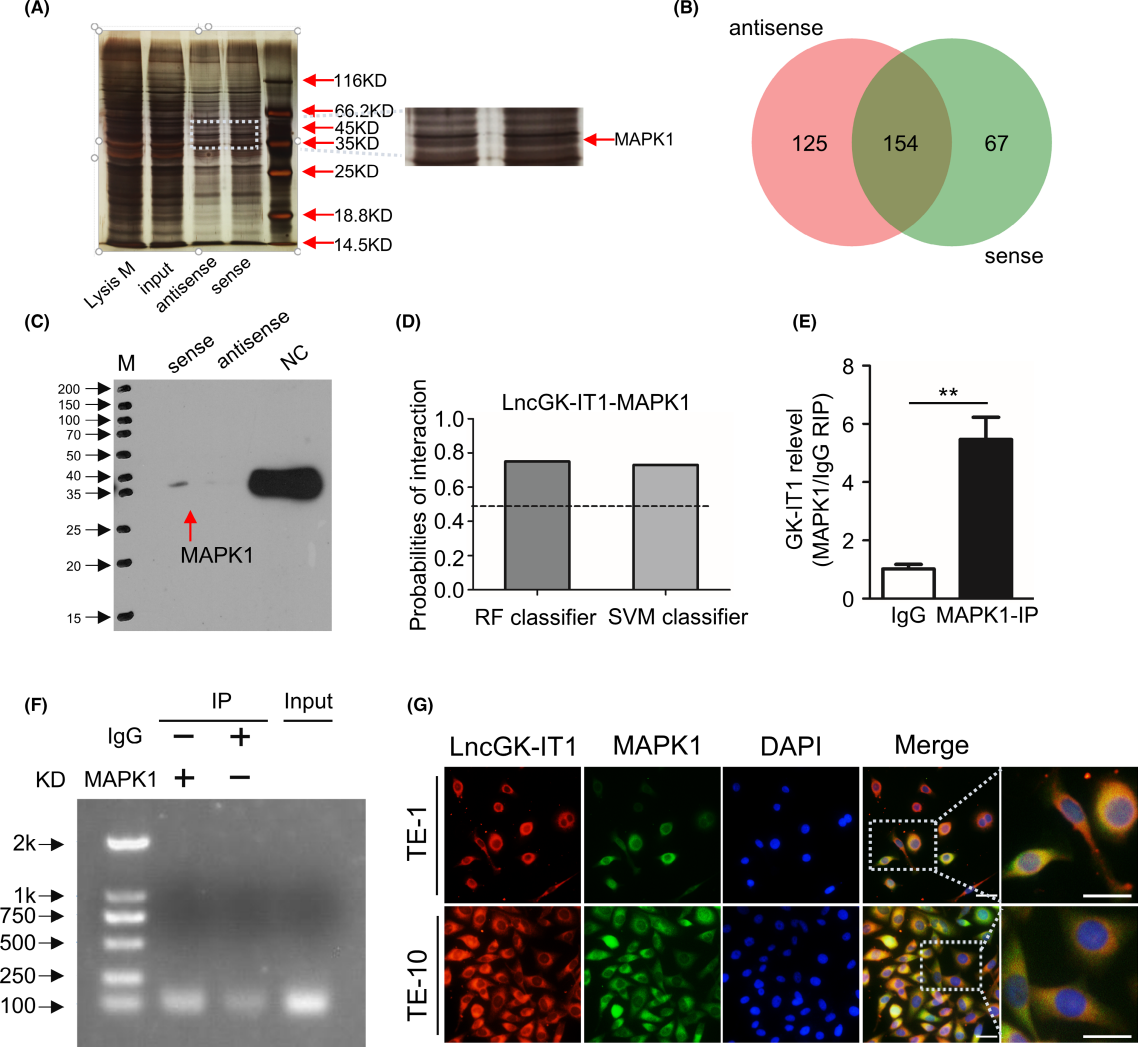
GK‐IT1 directly interacts with MAPK1. (A) Silver staining of biotinylated GK‐IT1‐associated proteins after RNA pulldown. Antisense was used as RNA control. (B) GK‐IT1‐bound proteins with cancer relation identified by mass spectrometry. (C) Western blotting of MAPK1 following pulldown of GK‐IT1 or negative RNA control (NC). (D) Bioinformatics analysis of the possibility of interaction between GK‐IT1 and MAPK1. (E) Cell lysates were immunoprecipitated with MAPK1 or IgG antibody. Expression levels of GK‐IT1 in either immunoprecipitates or cell lysates (input) of ESCC cells were measured by qRT‐PCR (mean ± SD, *n* = 3). (F) Expression level of GK‐IT1 in either immunoprecipitates or cell lysates (input) of ESCC cells were measured by agarose gel electrophoresis. (G) The co‐localization of GK‐IT1 and MAPK1 was observed in ESCC cells (magnification, ×400, Scale bar, 50 μm) by FISH and IF assay. The data are presented as the mean ± SD, *p < 0.05, **p < 0.01, ***p < 0.001

Previous results showed that GK‐IT1 was predominantly located in the cytoplasm (Figure 2K,L and Figure S1B). Thus, we focused on clarifying its potential functions with respect to cytoplasmic location in ESCC cells. Considering that lncRNAs are able to bind macro‐biomolecules in the cytoplasm,^28^ we performed an RNA pulldown assay and LC–MS analysis to detect potential interacting proteins of GK‐IT1 (Figure 5A). A total of 221 and 279 proteins were identified to interact with sense and antisense GK‐IT1 strands, respectively. The number of proteins exclusively associated with GK‐IT1 sense and antisense strands was 67 and 125, respectively (CI: 95%, unique peptides: 1; Figure 5B; Table S2). Among the 67 proteins that were pulled down with GK‐IT1 sense strands, MAPK1 attracted our attention because of the widely acknowledged role of ERK/MAPK signaling in human cancer progression. Therefore, we selected MAPK1 for further analysis. Consistent with previous results, western blotting using an antibody against MAPK1 verified the association of GK‐IT1 sense strand with MAPK1 in pulldown assays (Figure 5C). Moreover, the possibility of the interaction between GK‐IT1 and MAPK1 was predicted to be high by the RPIseq website (https://pridb.gdcb.iastate.edu/RPISeq/) using two algorithms (RF, SVM) (Figure 5D). In addition, the RIP assay was carried out to further validate the association between MAPK1 and GK‐IT1 in ESCC cells. The level of GK‐IT1 was detected using two methods including qRT‐PCR and agarose gel electrophoresis following a RIP assay. Indeed, immune precipitants containing MAPK1 were enriched for GK‐IT1 sense transcripts (Figure 5E,F). Furthermore, we also used FISH and IF experiments to visualize the co‐localization of GK‐IT1 and MAPK1 in the cytoplasm (Figure 5G). Together, these results illustrate that GK‐IT1 can interact with MAPK1 in ESCC cells.

### 3.5 GK‐IT1 promotes ESCC progression by competitively binding to MAPK1 to disrupt DUSP6‐mediated inactivation of the ERK/MAPK signaling pathway

**FIGURE 6:**
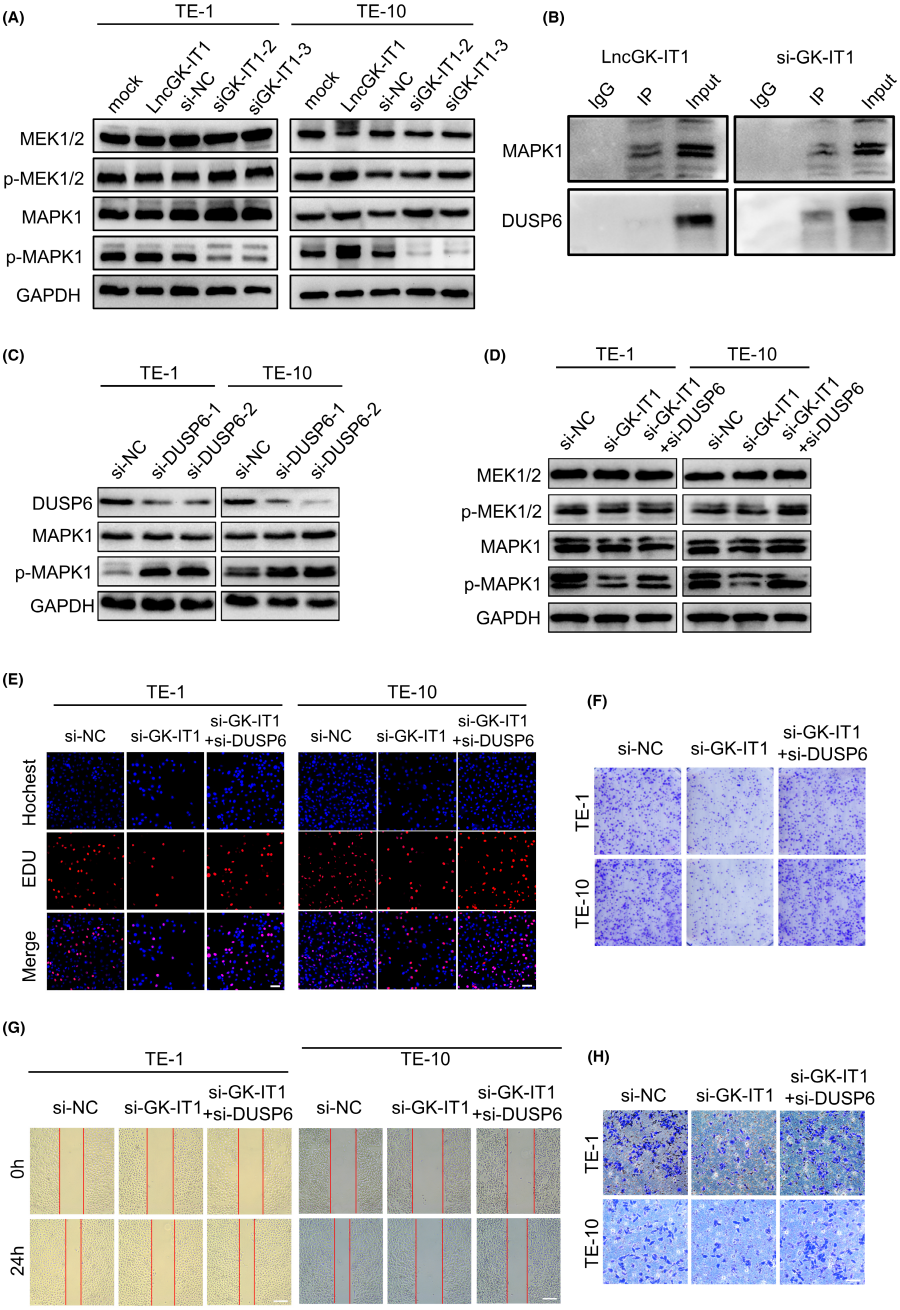
GK‐IT1 promotes ESCC progression via activation of ERK/MAPK signaling. (A) Western blot was conducted to determine the expression of proteins in the ERK/MAPK pathways in ESCC cells after transfection with si‐GK‐IT1 and overexpressing GK‐IT1. (B) Co‐IP assay showed that DUSP6 could not be pulled down by anti‐MAPK1 in TE‐10 cells with transfected with overexpressed LncGK‐IT1 plasmid but DUSP6 could be detected in TE‐10 cells with transfected with si‐LncGK‐IT1. (C) Knockdown of DUSP‐6 increased expression of ERK/MAPK‐related proteins in ESCC cells by western blotting. (D) The relative levels of proteins in ERK/MAPK pathway were measured in ESCC cells after transfection or co‐transfection with indicated si‐NC, siRNAs by western blot. (E) Cell proliferation was evaluated by EdU assay. (F) Cell proliferation was evaluated by colony formation assay. (G) The migration abilities of ESCC cells were measured by wound healing assays (magnification, ×50, Scale bar, 100 μm). (H) The invasive abilities of ESCC cells were assessed by transwell invasion assay (magnification, ×100, Scale bar, 100 μm)

Next, we sought to explore the potential role of GK‐IT1 in the activation of the ERK/MAPK signaling pathway by performing western blot. Notably, GK‐IT1 depletion significantly attenuated the phosphorylation of MAPK1, whereas overexpression of GK‐IT1 led to increased phosphorylated MAPK1 level. Meanwhile, the levels of both MAPK1 and phosphorylated MAP kinase‐ERK kinase 1/2 (p‐MEK1/2) remained unchanged upon silencing or overexpressing GK‐IT1 (Figure 6A and Figure S4A). MEK1/2 serves as the upstream kinase of MAPK1. These data indicated that GK‐IT1 could bind MAPK1 to promote the phosphorylation of MAPK1 in ESCCs. DUSP6 is a canonical negative regulator of ERK/MAPK signaling by inducing dephosphorylation of MAPK1.^29^ To clarify the potential mechanism underlying GK‐IT1‐mediated activation of ERK/MAPK signaling, a co‐immunoprecipitation assay was performed to measure whether DUSP6 was involved in the process. DUSP6 was not measured in the co‐immunoprecipitates of MAPK1 upon overexpression of GK‐IT1 (Figure 6B). On the other hand, GK‐IT1 knockdown led to notable increases of DUSP6 in the co‐immunoprecipitates of MAPK1 (Figure 6B). Silencing of DUSP6 significantly promoted the phosphorylation of MAPK1 (Figure 6C and Figure S4B,C). Moreover, DUSP6 depletion markedly abrogated the suppressive effects of GK‐IT1 knockdown on the activation of ERK/MAPK signaling (Figure 6D and Figure S4D). These data indicated that GK‐IT1 could promote activation of ERK/MAPK signaling by disrupting the interaction between MAPK1 and DUSP6.

Dysregulation of ERK/MAPK signaling has been reported to be involved in a variety of tumorigenic processes, including proliferation, survival, growth, and migration.^30^ To investigate whether the oncogenic role of GK‐IT1 could be attributed to the DUSP6/MAPK signaling axis, a series of functional experiments were conducted by inducing activation of the MAPK signaling pathway with knockdown of DUSP6 in GK‐IT1‐silenced ESCCs. Indeed, DUSP6 depletion significantly relieved the suppressive effects of GK‐IT1 knockdown on invasion and proliferation in ESCCs caused by GKI‐IT1 knockdown (Figure 6E–H and Figure S4E–H). Collectively, these data suggested that GK‐IT1 could competitively bind to MAPK1 to prevent the interaction between DUSP6 and MAPK1, thereby altering MAPK1 phosphorylation and promoting the malignant progression of ESCC.

### 3.6 GK‐IT1 facilitates tumor growth and metastasis in vivo

**FIGURE 7:**
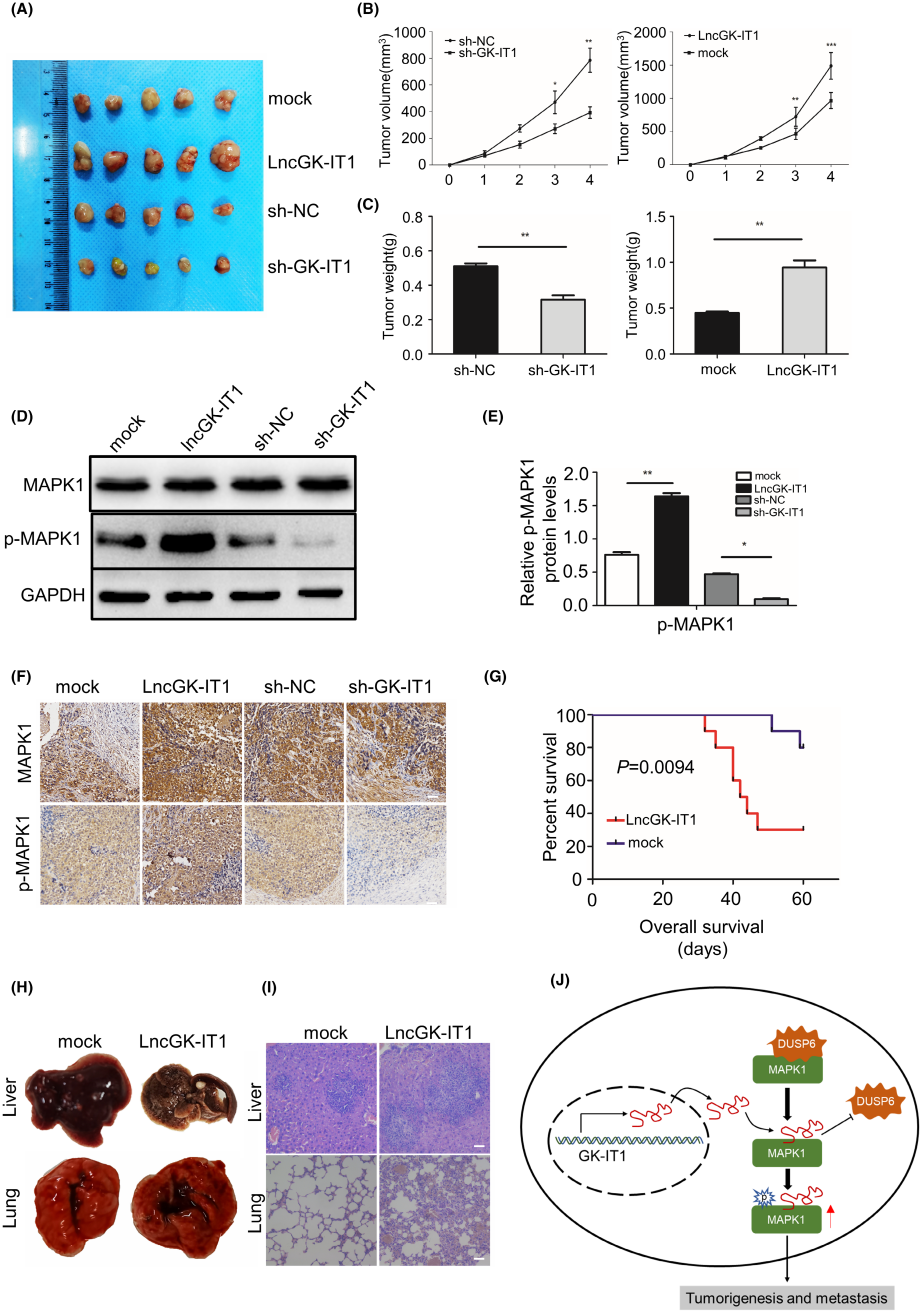
GK‐IT1 promotes oncogenesis and metastasis of ESCC in vivo. (A) The representative images of xenograft tumor in each group were displayed (*n* = 5). (B) Tumors volume was measured once a week to plot the growth curve. (C) Tumor weight was measured. (D, E) Western blot analysis was conducted to detect the protein level of MAPK1 and P‐MAPK1 in xenograft tumors. (F) The protein expression levels of MAPK1 and P‐MAPK1 in xenograft were verified by immunohistochemical experiment. (G) The survival curve of the nude mice injected with TE‐10 cells transfected with GK‐IT1 overexpressing or mock vector was drawn using Kaplan–Meier method. (H,I) H&E staining of the lungs (magnification, ×200, Scale bar, 100 μm) and livers (magnification, ×200, Scale bar, 100 μm) were showed. (J) The schematic diagram demonstrates the mechanism by which lncRNA GK‐IT1 promotes ESCC tumorigenesis and progression via antagonizing the binding of DUSP6 to MAPK1 to induce the activation of ERK/MAPK1 signaling. The data are presented as the mean ± SD, *p < 0.05, **p < 0.01, ***p < 0.001

To investigate the effects of GK‐IT1 on tumorigenesis in vivo, a xenograft tumor model of human ESCC was established. GK‐IT1‐overexpressing or GK‐IT1‐knockdown TE‐10 cells, and the corresponding control cells, were subcutaneously inoculated into female nude mice. The tumor burden in the GK‐IT1‐overexpressing group was notably higher than that in the control group, whereas depletion of GK‐IT1 significantly attenuated ESCC tumor growth (Figure 7A–C). Using western blotting experiments and IHC analysis, we found that the expression of GK‐IT1 was positively associated with elevated ERK/MAPK activity, as demonstrated by increased p‐MAPK1 (p‐ERK2) protein levels and unaltered total MAPK1 protein levels (Figure 7D–F). Moreover, the overall survival rate of nude mice injected with GK‐IT1‐overexpressing TE‐10 cells was strikingly lower than that of the control group (Figure 7G). Additionally, the metastatic liver and lung tumor burden in the GK‐IT1‐overexpressing group was higher than that of the control group, indicating that GK‐IT1 induced an enhanced metastatic ability in ESCCs in vivo (Figure 7H–I). In summary, these results indicated that GK‐IT1 promoted the growth and metastasis of ESCC in vivo.

## 4 DISCUSSION

ESCC generally results in a poor prognosis. Most ESCC patients present with tumor metastasis at initial diagnosis.^31^ Standard treatment approaches have remained largely unchanged over the past two decades, therefore, more efficient therapeutic strategy to improve the survival of ESCC patients is urgently needed.^32^ LncRNAs have emerged as key regulators of cancer‐related pathways, as well as biomarkers of various others diseases.^33^ However, the molecular mechanism of lncRNAs in the progression of ESCC is largely unknown. GK‐IT1, a long noncoding RNA (lncRNA), is located on human chromosome X: 30,671,635‐30,672,166 and transcripted from GK intronic region. The present study demonstrated that GK‐IT1 was aberrantly overexpressed in ESCC cells and tissues for the first time. The expression of GK‐IT1 was positively associated with tumor progression in ESCC patients. Functionally, we found that GK‐IT1 could facilitate tumor progression in vitro and in vivo. Mechanistically, GK‐IT1 could competitively bind to MAPK1 to prevent the interaction between DUSP6 and MAPK1, thereby governing the phosphorylation of MAPK1 and promoting the malignant progression of ESCC (Figure 7J).

LncRNAs play a vital role in cancer development and progression, including metastasis, therapy‐resistance, and immunosuppression.^34^, ^35^ For example, Wang et al. found that lncTUG1 enhanced the radiation resistance of ESCC by lowering miR‐1443p levels and regulating the MET/EGFR/AKT axis.^36^ The lncAMPC/LIF/LIFR axis has been shown to play a critical role in prostate cancer metastasis and immunosuppression.^37^ Over the past decade, therapeutic targeting of lncRNA for the treatment of cancer is gaining interest because of its diverse functional repertoire.^17^ Thus, it is of great importance to elucidate the oncogenic role and mode of action of lncRNAs in various cancers. A recent study demonstrated that GK‐IT1 was a potential diagnostic biomarker for gastric cancer through bioinformatics analysis.^38^ Our study demonstrated the clinical implication of GK‐IT1 in ESCC. In addition, we showed that GK‐IT1 could promote the proliferation and metastasis of ESCC cells for the first time.

LncRNAs exert their oncogenic roles through a variety of distinct mechanisms.^33^ It has been reported that lncRNAs could regulate gene expression through multiple mechanisms, such as epigenetic, transcriptional, and chromatin regulation.^39^, ^40^ For example, LNMAT2 epigenetically upregulates PROX1 expression by recruiting hnRNPA2B1 and increasing the H3K4 trimethylation level of the PROX1 promoter in bladder cancer.^41^ LncRNA MALAT1 binds and inactivates the prometastatic transcription factor TEAD, preventing TEAD from associating with its co‐activator YAP and target gene promoters in breast cancer cells.^42^ Moreover, some lncRNAs can combine with proteins and regulate their activity. For instance, lncRNA PSCA can interact with DDX5 and promote its degradation in gastric cancer.^43^ LncRNA KB‐1980E6.3 enhances the stability of c‐Myc mRNA by binding to IGF2BP1, therefore maintaining breast cancer stemness under hypoxic conditions.^44^ It has been reported that the interaction between lncRNA SPRY4‐IT1 and HNRNPL could mediate the TNF signaling pathway in HCC.^45^ However, the interaction between lncRNAs and proteins in ESCC has not been well‐studied. In our study, we identified several interacting protein partners of GK‐IT1 in ESCC cells through RNA pulldown and LC–MS analysis (Table S3). We therefore sought to investigate the relationship between GK‐IT1 and MAPK1 because of the widely acknowledged roles of ERK/MAPK signaling in human cancer progression.

The ERK/MAPK pathway is one of the main oncogenic pathways that induces uncontrolled proliferation, survival, and dedifferentiation of multiple cancer types.^46^ MAPK1, also known as ERK2, is the effector kinase of the ERK/MAPK pathway. MAPK1 can phosphorylate multiple substrates in different cellular compartments to regulate biological processes.^47^ Of note, the dephosphorylation and inactivation of ERK1/2 are catalyzed by phosphatases such as dual specificity phosphatases, protein‐tyrosine‐specific phosphatases, and protein‐serine/threonine phosphatases.^19^ As a member of dual specificity phosphatases subfamily, DUSP6 is tightly linked to malignant progression in various cancers.^48^ It has been reported that ΔNp63α directly bound to the promoter of the DUSP6 gene, regulating DUSP6 expression, and MAPK1 activation.^13^ Emerging evidence has shown that RNA molecules and proteins could affect the interaction between DUSP6 and MAPK1.^24^, ^25^ Consistent with these observations, we revealed that GK‐IT1 was involved in the aberrant activation of the ERK/MAPK pathway by attenuating the interaction between MAPK1 and DUSP6, thereby suppressing DUSP6‐mediated dephosphorylation of MAPK1 and facilitating the malignant progression of ESCC. Our findings advanced the understanding of the dysregulation of ERK/MAPK signaling by lncRNAs in cancer.

Taken together, this study demonstrated that dysregulation of lncRNA GK‐IT1 was involved in the aberrant activation of the ERK/MAPK pathway, which contributed to the development and metastasis of ESCC. Importantly, we provided novel evidence of the clinical relevance of GK‐IT1 in ESCC patients. To the best of our knowledge, this is the first time that GK‐IT1 has been reported to be implicated in the malignant progression of human cancers. Moreover, this study also highlighted a novel mechanism by which GK‐IT1 competitively bound to MAPK1 and attenuated the interaction between DUSP6 and MAPK1, leading to activation of the ERK/MAPK pathway. These findings offered new mechanistic understandings of ESCC progression, and thus may facilitate the development of new diagnostic indicators and targeted therapy approaches for ESCC.

There were several limitations to the present study. First, only 70 paired tissues from ESCC patients were collected, which may bias the results because of the relatively small sample size and heterogeneity among the patients. Second, we have only verified the overexpression of GK‐IT1 in ESCCs. However, the upstream mechanism contributing to the aberrant upregulation of GK‐IT1 in ESCC remains to be investigated. Third, the specific protein domains responsible for the interaction between MAPK1 and DUSP6 that were attenuated by GK‐IT1 warrant future studies.

## 5 CONCLUSION

To summarize, we identified GK‐IT1, an unreported lncRNA in ESCC, and demonstrated that GK‐IT1 expression was upregulated in ESCC tissues/cells and associated with poor prognosis in ESCC patients. Furthermore, we found that overexpression of GK‐IT1 could effectively increase ESCC cell proliferation, invasion, and metastasis. Mechanistically, we observed that GK‐IT1 could competitively bind to MAPK1 to prevent the interaction between DUSP6 and MAPK1, thereby governing the phosphorylation of MAPK1 and facilitating the malignant progression of ESCC. Our findings suggested that GK‐IT1 may represent a promising prognostic biomarker as well as a potential therapeutic target for ESCC.

## CONSENT FOR PUBLICATION

All authors give consent for the publication of the manuscript in Cancer Medicine.

## CONFLICT OF INTEREST

The authors declare that they have no conflict of interest.

## ETHICAL APPROVAL

The study was authorized by the Ethics Committee of The First Affiliated Hospital of Chongqing Medical University. All patients signed consent forms. The animal protocols were approved by Chongqing Medical University Animal Care and Use Committee (approval ID: 2020‐732).

## Supporting information


AppendixS 1

## Data Availability

The datasets used and analysed during the current study are available from the corresponding author on reasonable request.
