# Supplementary material for: Long noncoding RNA GK‐IT1 promotes esophageal squamous cell carcinoma by regulating MAPK1 phosphorylation
Source: Cancer Med. 2022 May 24;11(23):4555–74. doi: 10.1002/cam4.4795 (PMC9741976; doi:10.1002/cam4.4795)
Supplement: Supplementary file 1 — AppendixS 1 [file CAM4-11-4555-s001.zip › CAM4_4795_Additional file 2.docx]

**Table S1** primers and siRNA target sequences used in this study

| # qRT-PCR primers (5′-3′) | | | |
| --- | --- | --- | --- |
|  | Forward | | Reverse |
| GK-IT1 | | AGCGGTAGAGTCAGCTCTGTTTG | CACCATGCCCAGCCGTAAC |
| GAPDH | | GAAGGTGAAGGTCGGAGTC | GAAGGTGAAGGTCGGAGTC |
| U6 | | CTCGCTTCGGCAGCACA | CTCGCTTCGGCAGCACA |
| DUSP-6 | | GAAATGGCGATCAGCAAGACG | CGACGACTCGTATAGCTCCTG |
| # siRNA sequences (5′-3′) | | | |
| si-GK-IT1-1 | | CGUUGAGAGUCUUAGUAAA | UUUACUAAGACUCUCAACG |
| si-GK-IT1-2 | | GGAAGGUUCUGUAGCUAUA | GGAAGGUUCUGUAGCUAUA |
| si-GK-IT1-3 | | CUAUGAGUGUAUAGAGAAA | UUUCUCUAUACACUCAUAG |
| DUSP-6-si-1 | | AGCTCAATCTGTCGATGAA |  |
| DUSP-6-si-2 | | CCAACCAGAATGTATACCA |  |
| DUSP-6-si-3 | | GTGACTGTGGCTTACCTTA |  |
