# Supplementary figures and images for: Long noncoding RNA GK‐IT1 promotes esophageal squamous cell carcinoma by regulating MAPK1 phosphorylation
Source: Cancer Med. 2022 May 24;11(23):4555–74. doi: 10.1002/cam4.4795 (PMC9741976; doi:10.1002/cam4.4795)

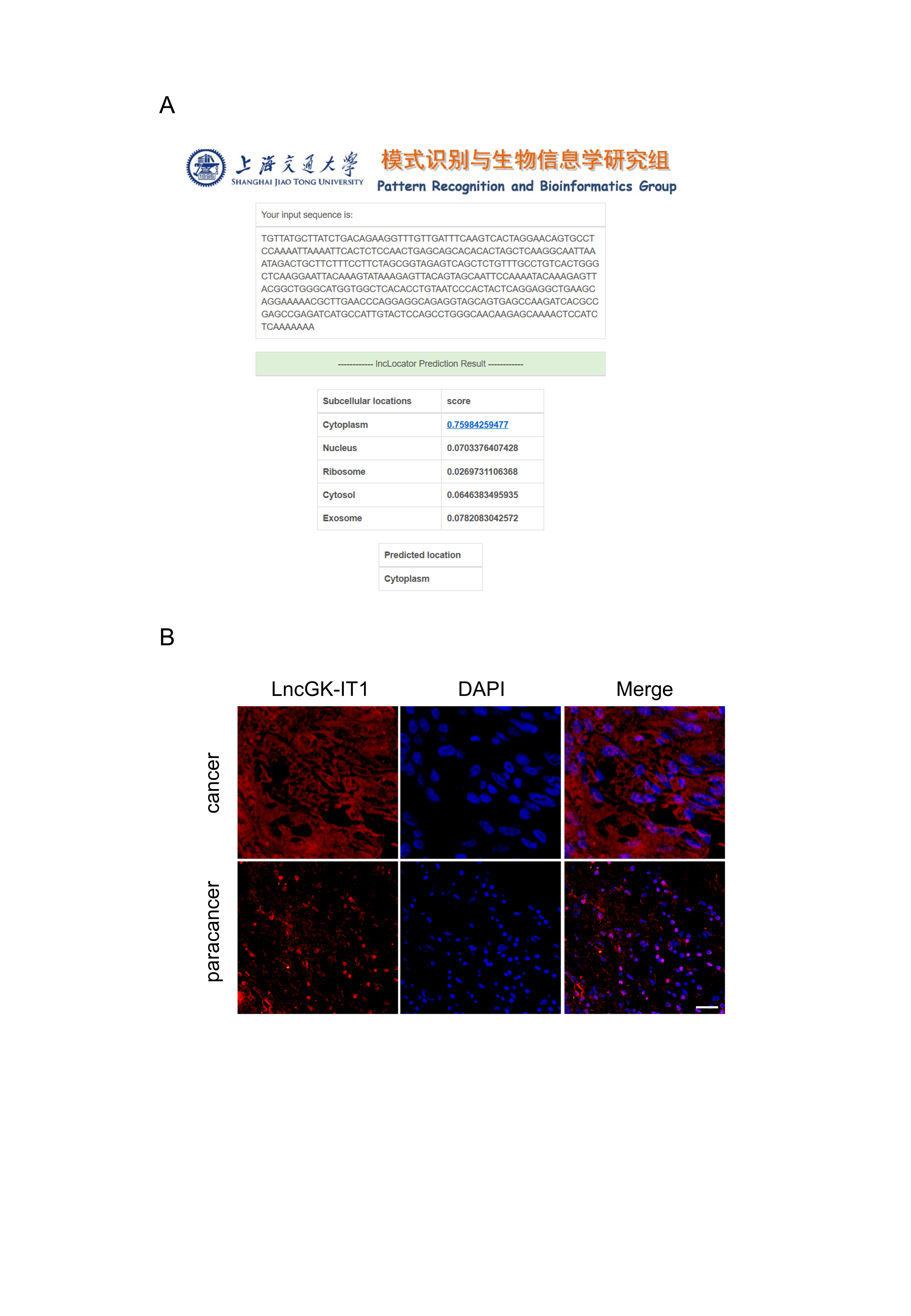

Supplement: Supplementary file 1 — AppendixS 1 [file CAM4-11-4555-s001.zip › CAM4_4795_FigureS1.TIF]

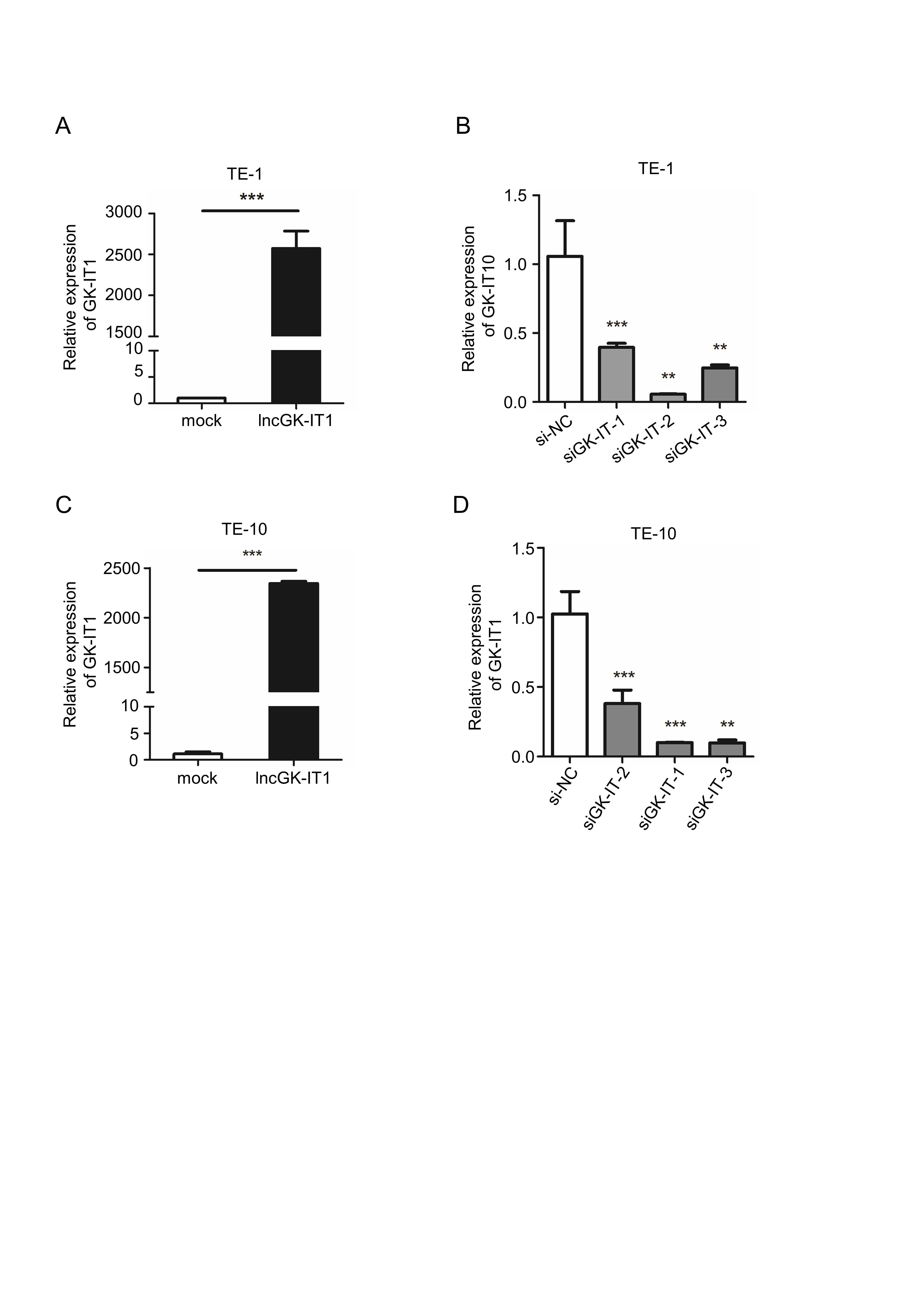

Supplement: Supplementary file 1 — AppendixS 1 [file CAM4-11-4555-s001.zip › CAM4_4795_FigureS2.TIF]

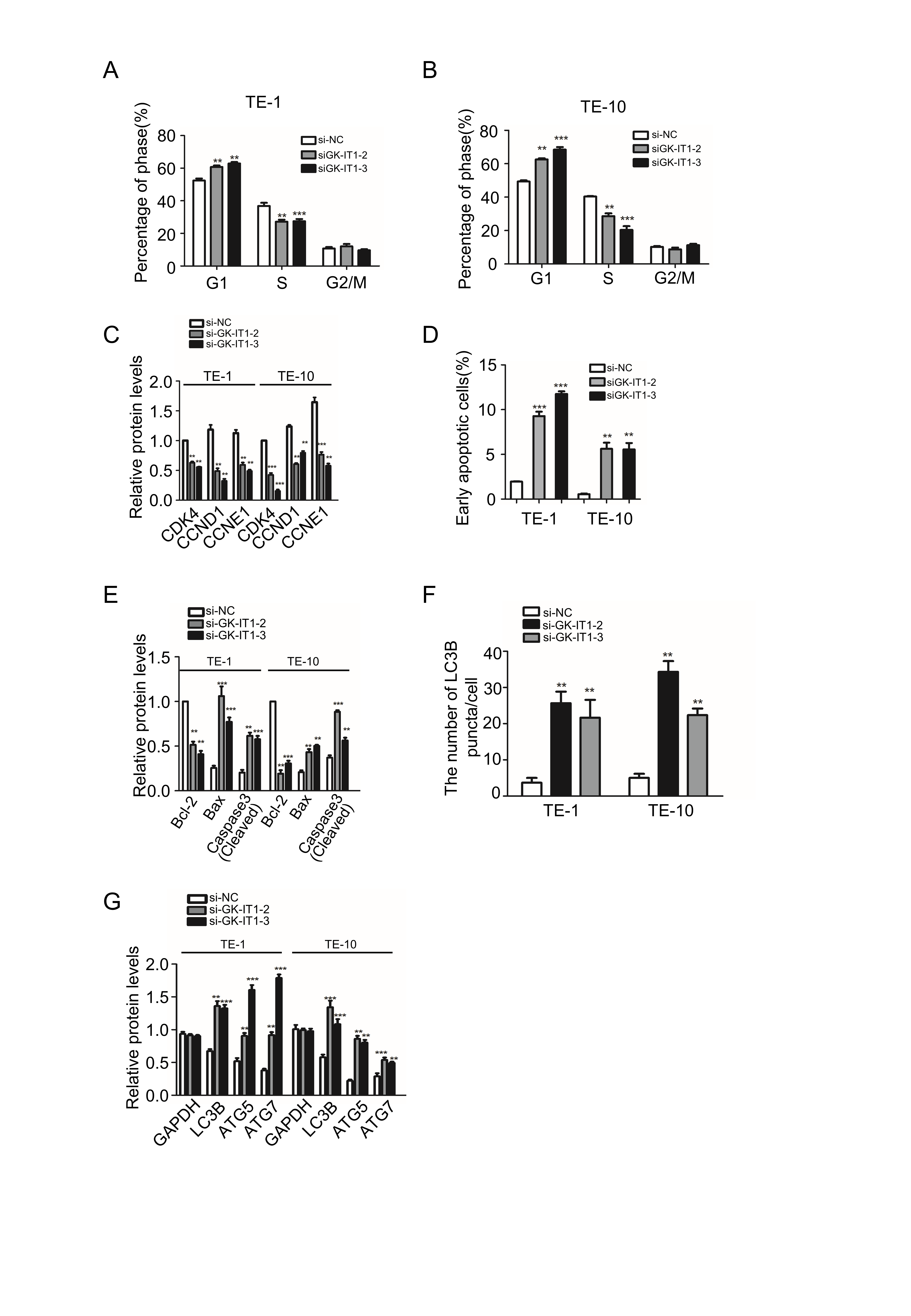

Supplement: Supplementary file 1 — AppendixS 1 [file CAM4-11-4555-s001.zip › CAM4_4795_FigureS3.TIF]

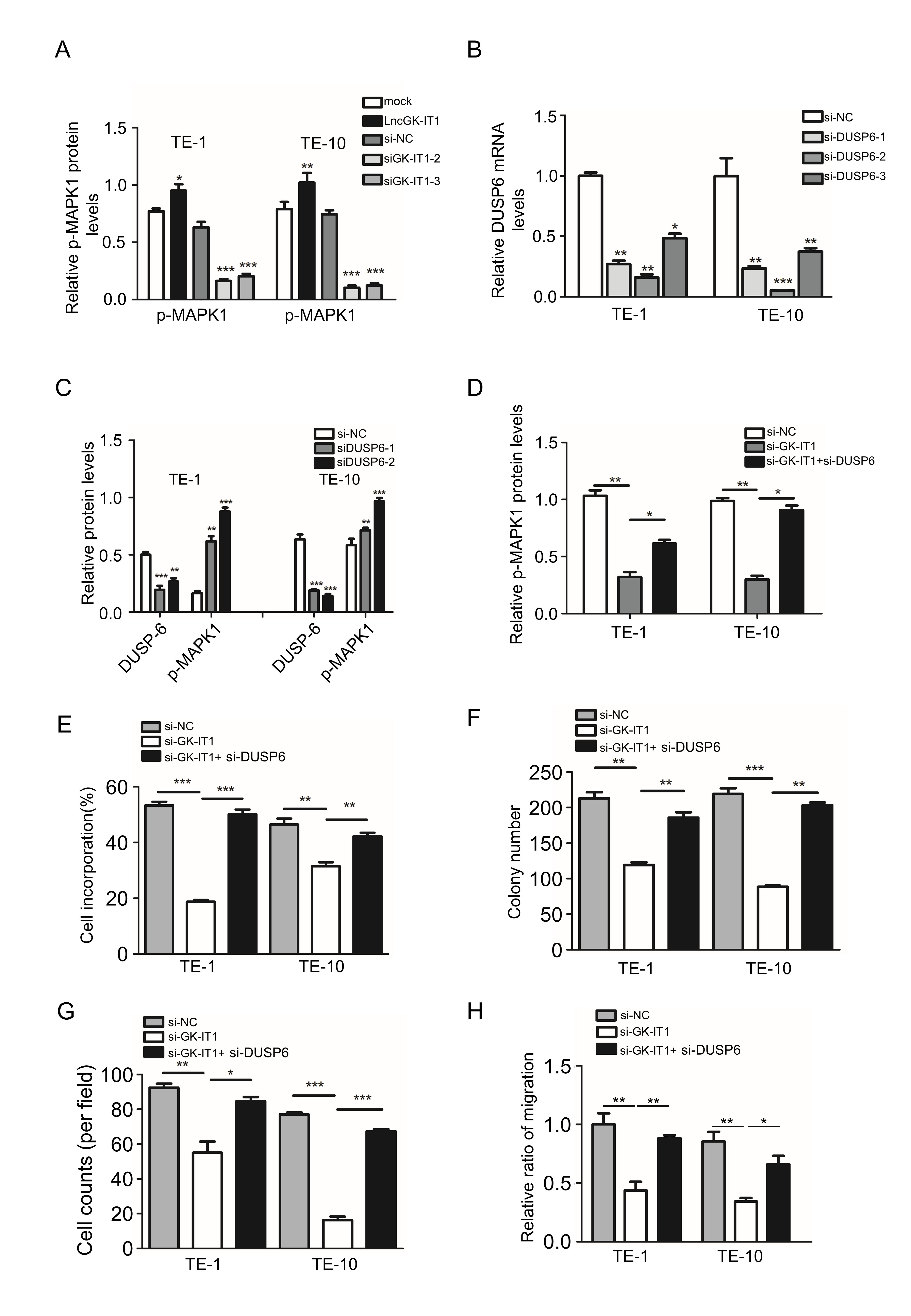

Supplement: Supplementary file 1 — AppendixS 1 [file CAM4-11-4555-s001.zip › CAM4_4795_FigureS4.tif]
